# Role of BMI1 in epithelial ovarian cancer: investigated via the CRISPR/Cas9 system and RNA sequencing

**DOI:** 10.1186/s13048-018-0406-z

**Published:** 2018-04-23

**Authors:** Qianying Zhao, Qiuhong Qian, Dongyan Cao, Jiaxin Yang, Ting Gui, Keng Shen

**Affiliations:** 1 Department of Obstetrics and Gynecology, Peking Union Medical College Hospital, Chinese Academy of Medical Sciences and Peking Union Medical College, No.1 Shuaifuyuan, Dongcheng District, Beijing, China; 2 Department of Gynecology and Obstetrics, West China Second University Hospital, Sichuan University, Key Laboratory of Birth Defects and Related Diseases of Women and Children (Sichuan University), Ministry of Education, Chengdu, China; 3 Department of Obstetrics and Gynecology, Qilu Hospital of Shandong University, Shandong, China

**Keywords:** B-cell-specific Moloney murine leukemia virus integration site 1 (BMI1), Clustered regularly interspaced short palindromic repeats (CRISPR)/Cas9, Epithelial ovarian cancer (EOC), Focal adhesion pathway, PI3K/AKT pathway

## Abstract

**Background:**

B-cell-specific Moloney murine leukemia virus integration site 1 (BMI1) might be an appropriate biomarker in the management of epithelial ovarian cancer (EOC). However, the biological role of BMI1 and its relevant molecular mechanism needs further elaboration. Clustered regularly interspaced short palindromic repeats (CRISPR)/Cas9 system is an excellent genome-editing tool and is scarcely used in EOC studies.

**Methods:**

We first applied CRISPR/Cas9 technique to silence BMI1 in EOC cells; thereafter we accomplished various in vivo and in vitro experiments to detect biological behaviors of ovarian cancer cells, including MTT, flow cytometry, Transwell, real-time polymerase chain reaction and western blotting assays, etc.; eventually, we used RNA sequencing to reveal the underlying molecular traits driven by BMI1 in EOC.

**Results:**

We successfully shut off the expression of BMI1 in EOC cells using CRISPR/Cas9 system, providing an ideal cellular model for investigations of target gene. Silencing BMI1 could reduce cell growth and metastasis, promote cell apoptosis, and enhance the platinum sensitivity of EOC cells. BMI1 might alter extracellular matrix structure and angiogenesis of tumor cells through regulating Focal adhesion and PI3K/AKT pathways.

**Conclusion:**

BMI1 is a potential biomarker in EOC management, especially for tumor progression and chemo-resistance. Molecular traits, including BMI1 and core genes in Focal adhesion and PI3K/AKT pathways, might be alternatives as therapeutic targets for EOC.

**Electronic supplementary material:**

The online version of this article (10.1186/s13048-018-0406-z) contains supplementary material, which is available to authorized users.

## Background

Epithelial ovarian cancer (EOC) is the leading cause of mortality among gynecologic cancers, and the majority of patients are diagnosed in advanced stages [1, 2]. Approximately, 70% of the patients would relapse even if they have received optimal cytoreductive surgery (CRS) combined with standard platinum-based chemotherapy. The 5-year survival rate for advanced-stage patients is very low [3]. It has been a major research focus and challenge to search for appropriate tumor markers and effective therapeutic targets because the etiology of EOC is largely unknown and the tumors present heterogeneity in multiple dimensions.

As a core member of Polycomb group proteins (PcG), B-cell-specific Moloney murine leukemia virus integration site 1 (BMI1) plays an important role in epigenetics, participates in important cellular events and is identified as aberrantly expressed in various human cancers [4–11]. In our previous study, we also found significant over-expression of BMI1 in metastatic lymph nodes and recurrent tumors compared to primary ovarian carcinomas. In addition, intensive expression of BMI1 in metastatic and recurrent tumors was an independent prognostic factor for survival and relapse, respectively [12]. However, the biological functions and related mechanism of BMI1 in EOC lack elaboration.

Among a variety of genome-editing techniques, the clustered regularly interspaced short palindromic repeats, (CRISPR)/Cas9 system has enormous prospects in tumor genesis and progression researches. The technique is highly effective at thoroughly shutting off gene expression with ~ 20 bp guide RNAs (gRNAs) [13, 14], by which can establish ideal cellular models for functional investigation. To date, studies involving the CRISPR/Cas9 technique in ovarian cancer studies are still scarce.

Therefore, we 1) explored the feasibility of the CRISPR/Cas9 system as a genome editing tool to construct BMI1 knock-out EOC cell models, 2) investigated the changes in biological behaviors after silencing BMI1 using in vitro and in vivo experiments, and 3) identified the differences in mRNA profiles between wild-type and BMI1 knock-out EOC cells using transcriptome sequencing technique in order to reveal the potential molecular mechanism. 4) revealed the potential of BMI1 serving as a biomarker in EOC management with basic research evidences.

## Methods

### Knocking out BMI1 in EOC cell lines using CRISPR/Cas9 technique

#### Cell culture

The ovarian cancer cell line SKOV3 was obtained from Institute of Basic Medical Sciences Chinese Academy of Medical Sciences & School of Basic Medicine, Peking Union Medical College. The SKOV3 cells were cultured in McCOY’ 5A medium (Macgene; Beijing, CN), supplemented with 10% fetal bovine serum (FBS, HyClone; Logan, UT, US), 100 U/ml penicillin (Solarbio; Beijing, CN), and 100 μg/ml streptomycin (Solarbio; Beijing, CN).

#### CRISPR/Cas9 vector construction and transfection

Specific gRNAs (Table 1) were designed using http://crispr.mit.edu/, and they were subsequently connected to linear pX330-BbsI vectors. To enhance editing efficiency, two gRNAs were synthesized targeting distinct domains of BMI1. Successively, two expression vectors (pX330-Cas9-gRNA1 and pX330-Cas9-gRNA2) were established, and co-transfected SKOV3 simultaneously with pLLexp-puro plasmid. Then, we used puromycin (1 μg/ml) to screen out those cells which have not been successfully transfected. Eventually, we cultured the viable cells surviving drug-sifting in 96-well plates using limited dilution method and amplified all the single-cell derived sub-clones.

**Table 1:** Sequences of gRNAs and primers

Name	Sequences (5′- > 3′)		PAM/ Length
	Forward	Reverse	
CRISPR gRNAs for BMI1			
gRNA1	CACCGAACGTGTATTGTTCGTT	AAACGGTAACGAACAATACACGTTC	ACC
gRNA2	CACCGTGGTCTGGTCTTGTGAAC	AAACAGTTCACAAGACCAGACCAC	TGG
Primers for PCR			
BMI1^a^	TTGATGCCACAACCATAATAGAAT CTAACACCAATGATTTATCCACTC	AATTACAAACAAGGAATTTCAACA	494 bp 194 bp
Primers for real-time PCR			
Caspase3	GAAATTGTGGAATTGATGCGTGA	CTACAACGATCCCCTCTGAAAAA	166 bp
Bcl-2	CTAAGGGTATGAAGGACCTGTA	CTCTGGAATCTAAAGGTCGT	111 bp
COL1A1	GTGCGATGACGTGATCTGTGA	CGGTGGTTTCTTGGTCGGT	119 bp
COL4A1	GGACTACCTGGAACAAAAGGG	GCCAAGTATCTCACCTGGATCA	240 bp
TNC	TCCCAGTGTTCGGTGGATCT	TTGATGCGATGTGTGAAGACA	131 bp
LAMA3	TGCTCAACTACCGTTCTGCC	TCCAGTTCTTTTGCGCTTTGT	181 bp
ITGA7	CAGCGAGTGGACCAGATCC	CCAAAGAGGAGGTAGTGGCTATC	203 bp
ITGB4	CTCCACCGAGTCAGCCTTC	CGGGTAGTCCTGTGTCCTGTA	133 bp
AKT3	AATGGACAGAAGCTATCCAGGC	TGATGGGTTGTAGAGGCATCC	130 bp
CREB5	AAAGACTGCCCAATAACAGCC	AAGCTGGGACAGGACTAGCA	88 bp
PIK3CA	GAAACAAGACGACTTTGTGACCT	CTTCACGGTTGCCTACTGGT	76 bp
PIK3CD	AGCCGGAAGACTACACGCT	GGTCAGGTGAGGGGTCAAC	122 bp
BIRC3	TTTCCGTGGCTCTTATTCAAACT	GCACAGTGGTAGGAACTTCTCAT	96 bp
GAPDH	ACAACTTTGGTATCGTGGAAGG	GCCATCACGCCACAGTTTC	101 bp

^a^Longer fragments obtained by the first BMI1 primer is more suitable for subsequent Sanger sequencing, whereas the second primer is better for revealing fragments shortening after silencing BMI1 on Agarose gel

#### Validation of BMI1 knock-out

Genomic DNA was extracted from each sub-clone for polymerase chain reaction (PCR) and Sanger sequencing of the targeted fragments. PCR primers are listed in Table 1. Protein was subsequently extracted from sub-clones with shortened nucleic acid sequences for western blotting. All validated BMI1 knock-out clones were then amplified and stored.

### Comparison of biological behaviors using in vitro and in vivo experiments

#### MTT assays

Cell viability was detected using MTT assays. Twenty microliters of MTT reagent (5 mg/ml) was added to each well (96-well plate) and incubated for 4 h and then terminated by adding 100 μl dimethylsulfoxide (DMSO) and incubating at 37 °C in the dark for 10 min. Cell proliferation was assessed by measuring the absorbance at 570 nm and 630 nm wavelength (Optical density, OD_570-630nm_). Growth curves were drawn with doubling time calculated for each group by consecutive MTT assays for seven days.

#### Flow cytometry (FCM)

The cell pattern of each sub-clone was detected using C6 FCM (BD Biosciences; New Jersey, US). Briefly, cell pretreatment comprised cold-ethanol fixation for 2 h and propidium iodide (PI, Keygene; Wageningen, the Netherlands) staining for 30 min in the dark. Modfit software (Verity; Maine, US) was used for analysis. Similarly, apoptosis could be compared by FCM after Annexin V-fluorescein isothiocyanate (FITC) and PI staining.

#### Transwell migration and invasion assays

The cell migration assay was performed using a Transwell chamber (BD Falcon™, San Jose, CA). These chambers were inserted into 24-well cell culture plates. Wild-type SKOV3 cells and BMI1 knock-out clones in 200 μl serum-free culture solution were added to the upper chambers. Ten percent FBS-containing McCOY’ 5A medium was added into the lower chambers to serve as the chemo-attractant. For invasion assays upper chambers were pre-coated with Matrigel (BD BioCoat™, BD Biosciences, San Jose, CA). After incubation for 24 h, the medium, the gel and uncrossed cells in the upper chambers were removed, while the migrated/invaded cells at the lower side of the membranes were fixed with paraformaldehyde (4%) and stained with crystal violet. Pictures were taken at 200X magnification, and cell numbers from five random microscopic fields were counted for statistical comparison.

#### Real-time PCR and western blotting

Expression levels of marker proteins in apoptosis were compared between wild-type and BMI1 knock-out cells by real-time PCR and western blotting. Primers and antibodies are listed in Tables 1 and 2, respectively.

**Table 2:** Antibodies

Name	Corporation	Dilution ratio
BMI1	Cell signaling	1:1000
Caspase3	Abcam	1:1000
Bcl-2	Abcam	1:500
COL1A1	Abcam	1:1000
COL4A1	Bioworld	1:500
TNC	Abcam	1:2000
LAMA3	Abcam	1:2000
ITGA7	Bioworld	1:500
ITGB4	Abcam	1:2000
AKT3	Proteintech	1:500
CREB5	Proteintech	1:500
PIK3CA	Abcam	1:1000
PIK3CD	Abcam	1:500
BIRC3	Abcam	1:1000
β-Tubulin	Bioworld	1:3000
β-Actin	Cell signaling	1:1000

Total RNA was isolated using Trizol reagent (Invitrogen; California, US). For quantitative analysis, mRNA levels of target sequences were compared: RNA was first retro-transcribed with random primers using TransScript First-Strand cDNA Synthesis Kit (TransGen; Beijing, CN), and then real-time PCR was carried out using TransStart Tip Green qPCR SuperMix (TransGen; Beijing, CN) with specific primers. The comparative Ct method was used to calculate the relative abundance of mRNA compared to GAPDH expression.

Harvested EOC cells were washed in phosphate buffer (PBS) and lysed in ice-cold cell lysis buffer with freshly added 0.01% protease inhibitor and then incubated on ice for 15 min. Cell debris was discarded after ultrasonic breaking and centrifugation at 14000 rpm for 10 min at 4 °C. Afterwards, the supernatant was run on a sodium-dodecyl sulphate (SDS)-PAGE gel, transferred to a polyvinylidene fluoride (PVDF) membrane, hybridized with specific antibodies, and developed using electrochemiluminescence (ECL) methodology.

#### Chemotherapeutic response

MTT assays were also used to estimate the cells’ sensitivity to cisplatin, carboplatin and paclitaxel (National Institutes for Food and Drug Control; CN). Each anti-neoplastic medicine was diluted at gradient concentrations and added to 96-well plates (6 wells per group per concentration). After incubation at 37 °C for 48 h, MTT assays were performed as previously described. With OD_570nm_ and OD_630nm_ detected, the growth inhibition ratio was calculated under each concentration for each group (Formula 1). Afterwards, the drug concentration at which 50% of the cells were prevented from proliferating was obtained using GraphPad Prism 5.0 software (California, US).

$$ \mathrm{Growth}\kern0.5em \mathrm{inhibition}\kern0.5em \mathrm{rate}=\left(1-\frac{\mathrm{Test}\kern0.5em \mathrm{well}\kern0.5em {\mathrm{OD}}_{570\hbox{-} 630\mathrm{nm}}-\mathrm{Blank}\kern0.17em \mathrm{well}\;{\mathrm{OD}}_{570\hbox{-} 630\mathrm{nm}}}{\mathrm{Lowest}\ \mathrm{Concentration}\ \mathrm{well}\ {\mathrm{OD}}_{570-630\mathrm{nm}}-\mathrm{Blank}\kern0.17em \mathrm{well}\;{\mathrm{OD}}_{570\hbox{-} 630\mathrm{nm}}}\right)\times 100\% $$

#### Xenografted tumor experiments

Female nude mice (BALB/c) were purchased from Beijing Vital River Laboratory Animal Technology Corporation (Beijing, CN). All mice were housed and fed under specific pathogen-free conditions in an institution approved by the Beijing Laboratory Animal Research Center. All studies were approved and supervised by Peking Union Medical College Hospital. All mice used were 5 weeks old when the experiments initiated. Six mice were assigned per group. Cells cultivated from different groups (2 × 10^7^ cells) were injected subcutaneously. To compare the ability of tumor formation in vivo, the transplanted tumors were checked and dimensioned every 2 days. Feeding was stopped at the same time for each group after 10 weeks. Tumor formation rate and average tumor size (Length×Width^2^ × 0.5) were calculated and compared.

### Investigation of underlying molecular mechanism using RNA sequencing

Total RNA was extracted from wild-type SKOV3 and BMI1 knock-out clones, and transcriptome libraries were subsequently constructed using a KAPA stranded mRNA-Seq Kit (KAPA Biosystems; Massachusetts, US). High-throughput sequencing was accomplished on a Hiseq2000 platform (Illumina; California, US). Bioinformatics analyses included estimation of gene-expression amount, clustering, GO (Gene ontology) and KEGG (Kyoto Encyclopedia of Genes and Genome). Differential gene-expression profiles were validated by real-time quantitative PCR and western blotting; corresponding primers and antibodies are listed in Tables 1 and 2. Eventually, potential pathways involving BMI1 in tumor genesis and progression were proposed.

### Statistical analysis

Biological behaviors and transcriptome profiles were compared between wild-type and BMI1 knock-out EOC cells. Each experiment was performed in triplicate assays and repeated at least three times. All values were expressed as the means ± SD (standard deviation). Statistical significance was determined using two-sided Student’s t test or Fish’s exact test, and a value of *P* < 0.05 was considered significant. Statistical analyses were performed using Statistical Package for the Social Sciences 20.0 (IBM; New York, US).

## Results

### Knock-out BMI1 in EOC cells using CRISPR/Cas9 system

**Fig. 1:**
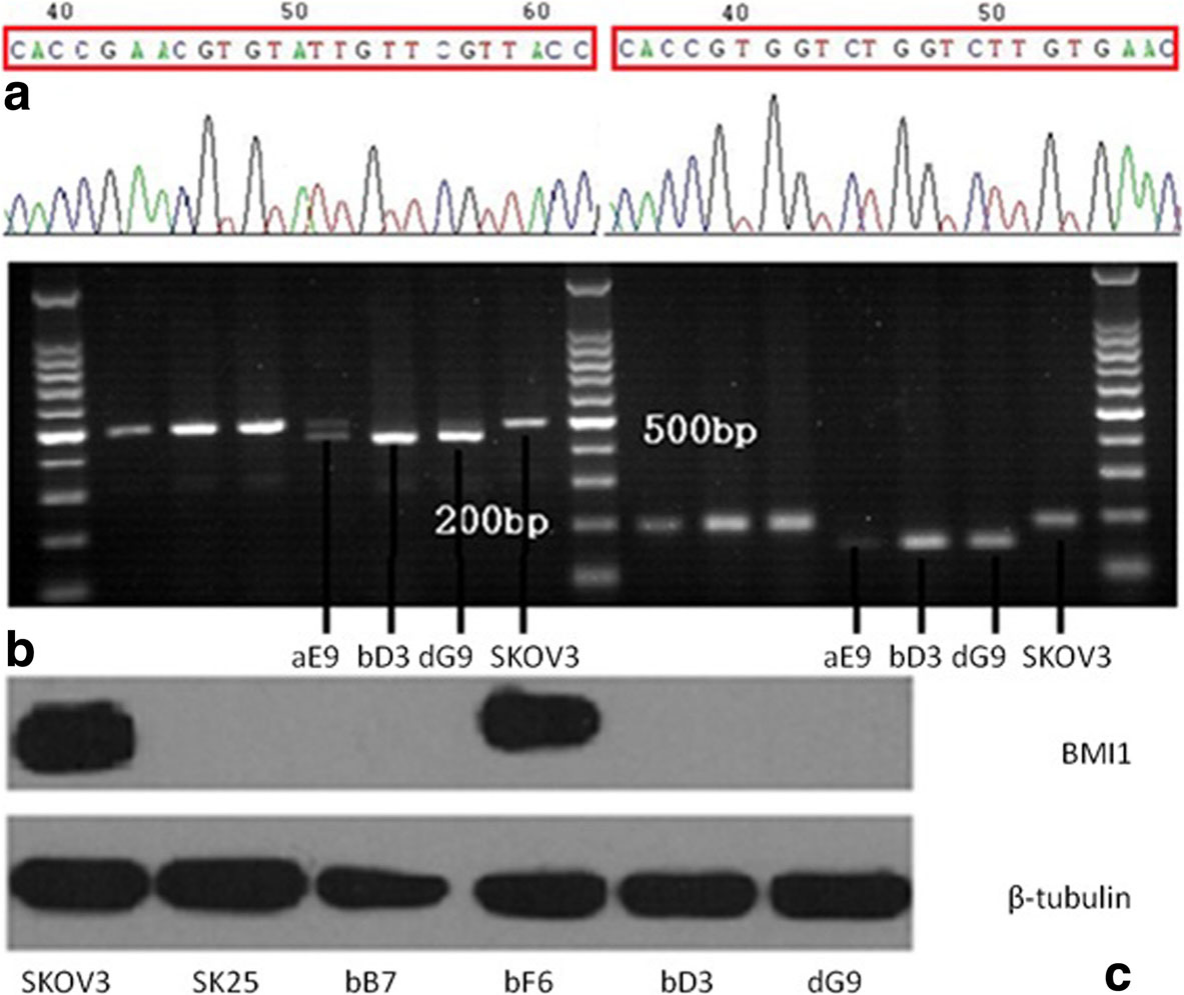
**a**. BMI1 gRNAs successfully inserted into pX330-BbsI vectors; **b**. Sub-clones with shortened nucleic acid sequences on Agarose gel after PCR; **c**. Sub-clones with no expression of BMI1 protein entirely detected by western blotting

Specific gRNAs were inserted into pX330-Cas9 vectors as shown in Fig. 1 with a success rate of 100%. After transfection and drug sifting, we obtained 23 single-cell derived BMI1 knock-out clones after limited-dilution culture. Figure 1 also depicts the validation results of target-gene editing: Agarose electrophoresis results revealed PCR products from the treated group had shortened nucleic acid sequences (9/23, Fig. 1b); target fragments of these sub-clones were confirmed deleted by Sanger sequencing (9/9); and western blotting experiments further verified that all nine sub-clones completely lack expression of BMI1 protein (Fig. 1c).

### Silencing BMI1 suppressed cell proliferation, migration and invasion of EOC cells

**Fig. 2:**
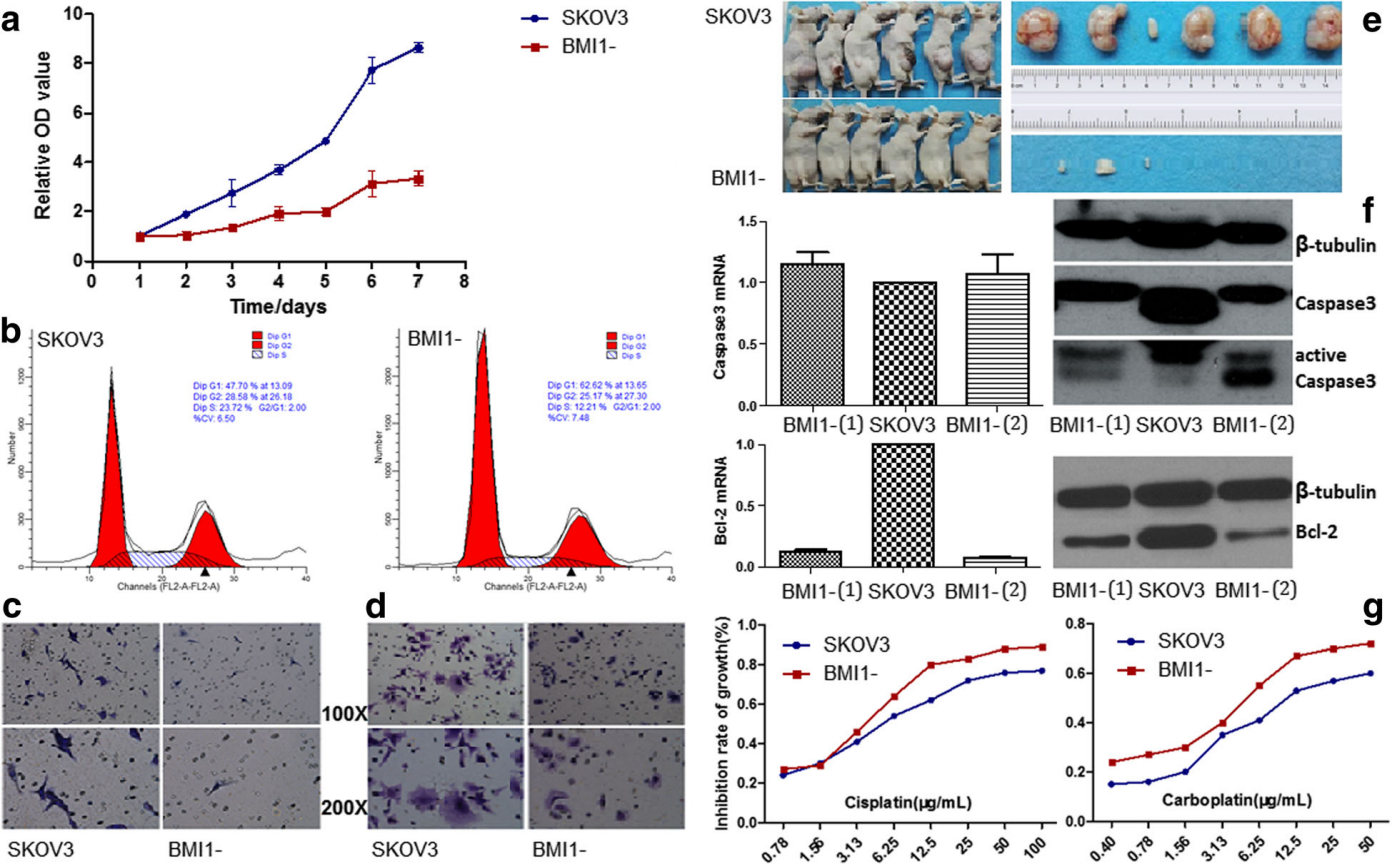
Changes of Biological behaviors after silencing BMI1: **a**. cell growth curve; **b**. cell cycle pattern; **c**. Transwell invasion; **d**. Transwell migration; **e**. in vivo experiments; **f**. apoptosis markers; **g**. chemotherapeutic responses

Growth curves showed that BMI1 knocking out reduced cell proliferation straight from the second through the seventh day, compared to the wild-type SKOV3 (Fig. 2a). The doubling times for BMI1 knock-out and wild-type clones were 2.10 ± 0.7 d and 5.62 ± 1.57 d, respectively (*P* < 0.05). BMI1 silencing also led to cell pattern alteration, as the proportion of EOC cells in S phase significantly decreased (23.7% vs. 12.2%, Fig. 2b). Cell invasiveness assessed using Matrigel-coated Transwell chambers presented a significant inhibition (Fig. 2c). Figure 2d shows reduced migration of SKOV3 cells transfected with CRISPR/Cas9-gRNAs. The numbers of migrated/invaded cells in either group are listed in Table 3. In addition, in vivo experiments (Fig. 2e) revealed that 1) tumor formation rate of subcutaneous inoculation reduced from 100% to 50% after BMI1 silencing and 2) the average xenografted tumor size was dramatically smaller in the BMI1 knock-out group (5.3 mm^3^) than in the wild-type group (1986.7 mm^3^).

**Table 3:** Results of Transwell experiments and IC50 for anti-neoplastic drugs

Group	Cell numbers (median ± SD)		IC50 (μg/ml)		
	Invasion	Migration	Cisplatin	Carboplatin	Paclitaxel
Wild-type SKOV3	9.2 ± 0.4	27.8 ± 3.2	5.9	14.5	16.6
BMI1 knock-out clone	4.2 ± 1.1	11.0 ± 0.7	3.4	5.2	13.6
*P* value	< 0.05	< 0.05	< 0.05	< 0.05	> 0.05

### BMI1 knock-out promoted apoptosis of EOC cells

We next examined the effect of BMI1 knocking out on cell apoptosis. First, FCM detected a higher apoptosis rate in BMI1 knock-out EOC cells than in the untreated group (3.3 ± 0.2% vs. 1.9 ± 0.2%, *P* < 0.05). Established markers of apoptosis include caspases. Therefore, we determined the expression of caspase 3 cleavages in control and BMI1-gRNAs-transfected EOC cells. An increase of active caspase 3 was observed in BMI1 knock-out cells (Fig. 2f). Bcl-2 suppresses apoptosis in a variety of cell systems, including functioning in a feedback loop system with caspases. We detected a remarkable reduction of Bcl-2 expression after knocking-out BMI1, indicating an ongoing apoptotic process (Fig. 2f, Additional file 1: Figure S1).

### Silencing BMI1 enhanced platinum sensitivity of EOC cells

We tested whether BMI1 silencing would affect chemotherapeutic responses of EOC cells. After drawing cell growth inhibition curves under gradient drug concentrations (Fig. 2g), IC50s of either sub-clone to cisplatin, carboplatin and paclitaxel were calculated (Table 3). The data demonstrated that BMI1 knock-out sensitized EOC cells to platinum medications (*P* < 0.05), including both cisplatin and carboplatin. However, similar reactions to paclitaxel were observed in BMI1-silenced and untreated cells (*P* > 0.05).

### BMI1 modulated focal adhesion and PI3K/AKT pathways in EOC cells

**Fig. 3:**
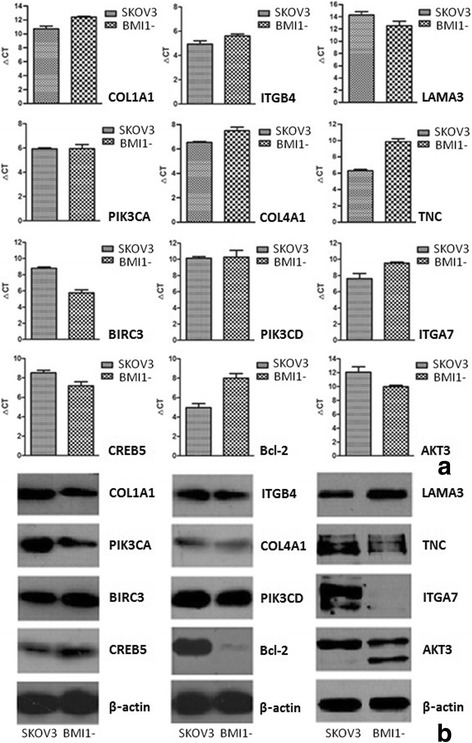
Validation of RNA sequencing results by **a**. RT-PCR; **b**. western blotting

Total RNA extracted from either group was qualified for RNA sequencing, revealing appropriate OD_260nm/280nm_ values (1.8–2.0) and abundances (≥5.0 μg per group). Raw reads were filtered and aligned to reference sequences. With GO analyses, we demonstrated that the majority of differentially expressed genes after BMI1 silencing participated in extracellular matrix (ECM) construction and blood vessel development. KEGG analyses revealed that BMI1 might alter the biological behaviors of EOC cells by modulating the focal adhesion and PI3K/AKT pathways. Expressions levels of related markers at both mRNA and protein levels were validated (Fig. 3): shutting-off BMI1 down-regulated transcription of COL1A1, COL4A1, TNC, ITGA7, ITGB4 and Bcl-2, while it up-regulated transcription of AKT3, LAMA3, CREB5 and BIRC3. Western blotting experiments on translational aspects showed results consistent with mRNA levels, except for additional under-expression of PIK3CA protein.

## Discussion

The CRISPR technique traces back to 1987, when scientists discovered corresponding reverse sequences following certain DNA fragments at the terminus of a bacterial genome [15, 16]. Between these short palindromic repeats were random DNA sequences (~ 30 bp), which were demonstrated later to be complementary to phage sequences [17, 18]. Recently, this technique has been generating excitement for its ability to modify genetic information rapidly and thoroughly. Compared to traditional techniques (e.g. RNA interfering, transcription activator-like effector nuclease), CRISPR/Cas9 system possesses several advantages: 1) the gRNAs are easy to design and stable in double-chain form; 2) target sequences are directly cut on DNA level, inducing an entire shut-off of gene expression; 3) multiple genes could be edited simultaneously, and DNA sequences could be knock-in as well as knock-out [13, 14]. We have successfully applied the CRISPR/Cas9 system and established a stable EOC cell model with BMI1 silenced entirely for further investigations.

BMI1 protein is one component of the polycomb repressive complex 1 (PRC1), which catalyzes lysine 119 mono-ubiquitination of histone H2A (H2AK119Ub1). H2AK119Ub1 is thought to contribute to gene silencing through the induction of chromatin compaction and inhibition of transcriptional elongation [19–21]. BMI1 has a broad impact on a diversity of cellular events: it controls the cell cycle by regulating the tumor suppressor proteins p16^INK4a^ and p14^ARF^ [22]; it promotes cell proliferation by suppressing the p16^INK4a^/retinoblastoma and/or the p14^ARF^/MDM2/p53 pathways [10]; it bypasses senescence and immortalizes cells by inducing telomerase activity in adult stem cells [9]; and it contributes to tissue homeostasis by maintaining self-renewal of hematopoietic, neural, prostate, intestinal, lung epithelial and bronchoalveolar stem cells [5, 23]. More importantly, accumulating genetic and epigenetic evidence has revealed BMI1, serving as a cancer stem cell marker, plays a crucial role in tumor heterogeneity and relapse [6, 24]. A growing number of recent studies have confirmed the oncogenic activation of BMI1 in diverse human malignancies and have explored the function of BMI1 as a pathway regulator in both stem cells and cancer cells [25, 26]. We discovered a remarkable inhibition of cell proliferation from silencing BMI1, with more EOC cells staying in G1 phase. A recent study has demonstrated that the activity of the estrogen receptor α (ERα)-coupled BMI1 signature impacts p16^INK4a^ and cyclin D1 status and correlates with the tumor molecular subtype and biologic behavior in breast cancer [9]. In addition, knocking out BMI1 reduced both the invasion and migration abilities of EOC cells. In accordance, other studies found that BMI1 was involved in inducing epithelial mesenchymal transition, leading to tumor invasion and metastasis [26]. Moreover, over-expression of BMI1 in EOC cells was found to up-regulate the expression of cyclin D1, CDK4 and Bcl-2, promoting cell growth and inhibiting cell apoptosis [27]. In contrast, silencing BMI1 in our study manifested a reverse impact on regulation of the cell cycle and apoptosis. Last but not the least, we found that knocking out BMI1 enhanced the platinum sensitivity of EOC cells. Similarly, Wang et al. revealed that down-regulating BMI1 increased the amount of reactive oxygen species, stimulated the DNA damage repair pathway, and eventually promoted the cisplatin-induced apoptosis [28]. Altogether, these evidences might shed light upon the most vexing conundrum in EOC management: chemo-resistance.

The CRISPR/Cas9 technique has provided an excellent cell model for analysis of one single variable: BMI1. We further investigated the most relevant genes using RNA sequencing. Most genes differentially expressed after silencing BMI1 were engaged in tissue development, ECM organization and blood vessel development, which might alter the microenvironment and angiogenesis of tumors, and then lead to transformation of malignant phenotypes: decrease in COL1A1 and COL4A1 might redress the reconstruction disorder of ECM [29, 30]; regulation of TNC and LAMA could increase tissue tension, reduce invasion and prevent migration of ovarian cancer cells [31, 32]. However, the clinical significance of these biomarkers in EOC management still needs validation because of controversial results from different studies. Furthermore, KEGG analyses revealed that the focal adhesion and PI3K/AKT pathways were involved after silencing BMI1. Aberrant expression of focal adhesion kinase (FAK) was related to the uncontrolled proliferation, suppressed apoptosis, invasion, angiogenesis and immune-depression of tumor cells [33]. One meta-analysis demonstrated that up-regulation of FAK indicated shorter overall survival in a variety of human cancers with a pooled hazards ratio of 1.815 and site specificity [34]. A growing number of studies have proved the activation of the PI3K/AKT/mTOR pathway in EOC [35–38], although the Cancer Genome Atlas research detected a low mutational rate (< 5%) of PI3KCA, AKT and PTEN in high-grade serous ovarian carcinomas (HsOCs) [39]. Other research found that PI3K genes were frequently mutated in non-HsOCs, which were relatively insensitive to platinum [38]. Another promising thing is that diverse anti-neoplastic drugs targeting the PI3K/AKT/mTOR pathway are under clinical investigations [39, 40].

## Conclusions

It is feasible to use the CRISPR/Cas9 system to construct ideal EOC cell models with target gene expression thoroughly shut off. Silencing BMI1 can change various biological behaviors of EOC cells, including reducing cell proliferation, migration, invasion, and promoting cell death; BMI1 might be a promising predictor for platinum sensitivity. BMI1 may accelerate tumor genesis and metastasis through dysregulating tumor ECM and blood vessel assemblies. Molecular traits, including BMI1 and crucial genes in the focal adhesion and PI3K/AKT pathways, might be potential biomarkers and novel therapeutic targets for EOC.

## Additional file

Additional file 1: **Figure S1.** PARP expression detected by western blotting. (PNG 29 kb)
