# Supplementary figures and images for: Role of BMI1 in epithelial ovarian cancer: investigated via the CRISPR/Cas9 system and RNA sequencing
Source: J Ovarian Res. 2018 Apr 23;11:31. doi: 10.1186/s13048-018-0406-z (PMC5911954; doi:10.1186/s13048-018-0406-z)

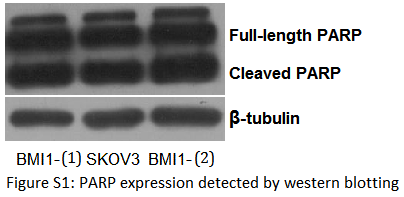

Supplement: Supplementary file 1 — Figure S1. PARP expression detected by western blotting. (PNG 29 kb) [file 13048_2018_406_MOESM1_ESM.png]
